# “It’s Like Listening to the Radio with a Little Interference”: A Qualitative Study Describing Pain Management among Patients with Psoriatic Arthritis

**DOI:** 10.3390/jcm12237348

**Published:** 2023-11-27

**Authors:** Nina Brodin, Björn Sundström, Mathilda Björk, Emma Swärdh

**Affiliations:** 1 Division of Physiotherapy, Department of Neurobiology, Health Care Sciences and Society, Karolinska Institutet, 14183 Huddinge, Sweden; emma.swardh@ki.se; 2 Division of Physiotherapy, Department of Orthopaedics, Danderyd Hospital, 18288 Stockholm, Sweden; 3 Centre for Research and Development, Uppsala University, Region Gävleborg, 80188 Gävle, Sweden; bjorn.sundstrom@umu.se; 4 Unit of Rheumatology, Department of Public Health and Clinical Medicine, Umeå University, 90187 Umeå, Sweden; 5 Pain and Rehabilitation Center, Department of Health, Medicine and Caring Sciences, Linköping University, 58183 Linköping, Sweden; mathilda.bjork@liu.se

**Keywords:** pain, psoriatic arthritis, rehabilitation, qualitative research

## Abstract

Pain is one of the most important areas to focus on in the assessment and treatment of psoriatic arthritis (PsA), and treatment should be individualized and based on the needs of the patient. Therefore, our aim was to explore and describe the management of pain among patients with PsA. We conducted semi-structured interviews with 11 participants with PsA (3 men and 8 women) and used qualitative content analysis to analyze the text. The results showed a main overarching theme of meaning and three subthemes. They were ‘Taking charge of life despite the constant murmur of pain’ through ‘Sorting out vulnerability’, ‘Reaching acceptance and engagement’, and ‘Directing focus to change’. Nine categories further described the components of pain management: ‘face uncertainty for the future, ‘consider restrictions’, ‘illuminate the invisible’, ‘increase awareness’, ‘find a permissive environment and social support’, ‘enhance inner endurance’, ‘reformulate emotions and thoughts’, ‘use distracting activities’, and ‘adjust activities’. The action components of pain management interpreted from a theoretical perspective highlight the importance for the patients of attaining the satisfaction of three basic psychological needs, i.e., competence, autonomy, and relatedness. Health professionals therefore need to increase the skills required for needs-supportive behaviors as well as facilitating spouse and peer support in the management of pain in PsA.

## 1. Introduction

Psoriatic arthritis (PsA) is an inflammatory rheumatic disease, which is associated with the skin disease psoriasis. In addition to skin involvement and joint inflammation, periarticular structures are often affected, which leads to a high incidence of inflammation in tendons and ligaments as well. Psoriatic arthritis is as common in men as in women and the age of onset is usually between 30 and 55 years [1]. As with several other inflammatory rheumatic diseases, the cause of the disease is unclear, but various components are assumed to contribute, e.g., genetic, immunological, and environmental factors.

People with PsA have a significantly worse health-related quality of life than the general population, something that has been suggested to be due to having two chronic diseases at the same time, a skin disease that causes cosmetic changes and a joint disease that causes functional limitations [2]. When compared to people with only skin psoriasis, the health-related quality of life and functional and activity capacity are more impaired in PsA [3,4,5,6,7].

Patients with psoriasis and PsA experience various psychosocial impacts of their disease, such as uncontrollable and ongoing upheaval, mental load, shame and judgment, inadequacies, and burden of therapy but also a need to gain control and make confident decisions regarding treatment [8]. Pain is common in people with PsA and occurs in the skin as well as in the structures of the musculoskeletal system [9,10]. Both international collaborations [11] and single studies that studied outcome measures, disease consequences, and experiences of pain in PsA emphasize that pain is one of the most important areas to focus on in the assessment and treatment of this patient group [12,13,14].

When pain becomes long-standing, as for many people with PsA, treatment should be individualized and based on the needs of the patient [15]. The experience of this long-standing pain is complex and consists of several components, including sensory, emotional, cognitive, behavioral, and social aspects. The pain often has negative social consequences for family life, interests, work, lifestyle, finances, and faith in the future [16,17]. When managing pain, sufficient coping strategies are identified as important [18] and international recommendations highlight that education and self-management support are key steps in non-pharmacological treatment [15]. Managing long-standing pain also requires the use of behavioral change components such as continuous support with booster sessions either from health professionals or peers, as well as increased knowledge of pain and the use of problem-solving techniques [19,20]. Thus, patients with long-standing pain need to adhere to and maintain self-management strategies to manage pain; however, this effort can be exhausting, and motivation can be difficult to sustain over time [20]. Motivation is, therefore, a vital part of the self-management of chronic pain where a patient’s willingness to engage in pain management is more likely to occur if the behaviors are self-determined and the motivation intrinsically determined [21].

To the best of our knowledge, no qualitative studies exploring the management of pain from the perspectives of patients with PsA exist. To address the needs of these people and enhance therapeutic relationships, the aim of the present study was thus to qualitatively explore and describe the management of pain among patients with PsA.

## 2. Materials and Methods

A descriptive design with a qualitative, inductive approach was applied, and conventional content analysis was used to describe the phenomenon systematically [22].

Fourteen participants were chosen with a purposeful sampling method aiming to include both men and women as well as to represent variations in age, sex, disease duration, activity limitation, and pain and fatigue intensity. The criteria for inclusion in the study were age above 18 years and a diagnosis of PsA confirmed at least 6 months previously. The criteria for exclusion from the study were cognitive impairments or language difficulties. The participants were recruited from an outpatient rheumatology specialist clinic in the central region of Sweden as well as from a university hospital in the northern region of Sweden. Patients fulfilling the inclusion criteria were approached for participation by their nurse at attendance at the clinic for a routine appointment. The first author, N.B., contacted all interested patients and provided oral information about the study, asked the patients to participate, and arranged a suitable time for conducting the data collection. Three of the participants did not come to the scheduled interview or could not be reached to book an interview appointment. Thus, eleven participants gave written consent and participated in interviews. The participants were informed that they were free to withdraw from the study at any time without giving any reason and confidentiality was guaranteed. Ethical approval was obtained from the Swedish Ethical Review Authority (Dnr 2018/2205-31-2 and 2020-03696).

Semi-structured interviews were conducted during 2019–2020 using an interview guide consisting of the main areas of inquiry (Table 1). The interview guide was subjected to discussion within the research team (N.B. and E.S.), and the final version was determined by the research team by reviewing the primary aim of the study and ensuring that the main areas of inquiry were appropriate for the aim. The interviews were performed by the first author (N.B.) and there was no relationship between the interviewer and the participants prior to the data collection. Open-ended questions were used along with follow-up questions to facilitate elaboration and elicit more details from the participants. Follow-up and probing questions varied slightly between participants because they were adjusted to individual responses during the interview. To minimize bias and enhance convenience, the participants chose the setting of the interview, conducted at the clinic, the research center, their home, or by telephone. All descriptive demographic data (Table 2) were collected from the participants before the interviews using a questionnaire specifically developed for the study, and data on pain, fatigue, general health, and activity limitations were collected at the same time using valid questionnaires. For participants who chose to be interviewed by telephone, all demographic and health-related data were collected through an interview with the first author (N.B.). Interviews lasted 24–59 min (median 36 min) and were audio-recorded and transcribed verbatim.

A qualitative inductive content analysis at a high abstraction level and with a high degree of interpretation was conducted, influenced by Lindgren et al. [23], as well as Elo and Kyngäs [22]. The following steps were used in the analysis: familiarization with the data, open coding and identification of parts in the interview relating to these codes, creation of categories, creation of descriptive subthemes, refining and labeling of subthemes, creation of a theme of meaning and selecting quotes for illustration purposes (Table 3). To ensure trustworthiness, several members of the research team took part in the analysis. The researcher (E.S., Ph.D.), who performed the primary analysis, and the researcher (N.B., Associate Professor) who both performed the interviews and re-analyzed the analysis and results were female, experienced physiotherapists and researchers in rheumatology, behavioral medicine, physical activity, and with expertise in both qualitative and quantitative research approaches. These two researchers also refined and relabeled the themes by discussing and comparing the emerging results and interpretations with the content of the original interviews until a consensus was reached. M.B. (Professor), an experienced female occupational therapist and researcher in rheumatology, chronic pain, pain management, and with expertise in theoretical perspectives on disability in a biopsychosocial framework of pain peer-reviewed the analysis.

## 3. Results

**Figure 1:**
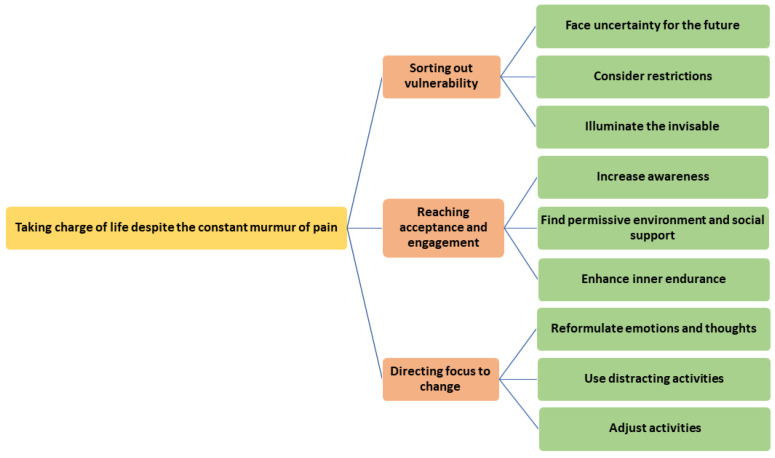
Management of pain among patients with PsA.

A main overarching theme of meaning ‘Taking charge of life despite the constant murmur of pain’ with three descriptive subthemes: ‘Sorting out vulnerability’, ‘Reaching acceptance and engagement’, and ‘Directing focus to change’, were identified as the participants’ management of pain (Figure 1). The overarching theme of meaning can be understood as the participants’ overall approach and goal for the management of pain, and the descriptive subthemes can be considered as ways to reach this goal. The descriptive subthemes are further illuminated through nine categories that describe important action components of the patients’ pain management.

### 3.1. Sorting out Vulnerability

#### 3.1.1. Facing Uncertainty about the Future

Experiencing vulnerability through apprehensive thoughts of pain facilitated both discomfort and feelings of uncertainty about the future in the participants. It was highlighted that this triggered both insecurity and fear about the future, e.g., not being able to play with grandchildren or total stiffness. The uncertainty was sometimes a mental battle and some participants described it as a feeling of living with massive tension and dark thoughts in the beginning after a diagnosis. Others expressed how anxiety and stress had created thoughts of ‘now or never’. Fear of ending up in a state where pain was uncontrollable or being hospitalized could sometimes appear and this was also a mental burden. Furthermore, a sense of being very fragile was present and some participants described a process of exaggerating small problems with pain into something much bigger than it was. Also, the feeling of not knowing how long a better period with less pain would last was sometimes present. Some participants expressed concerns about whether the pain would finally break them or not.

#### 3.1.2. Considering Restrictions

Limiting activities in daily life, decreasing social activities, as well as meeting mental challenges were highlighted as part of the management of pain that had to be considered in the participants’ lives. Daily activities such as climbing stairs, standing up, carrying children, as well as performing different household tasks were all affected by pain and this in turn influenced the everyday puzzle. Furthermore, decreased patience and sleep patterns as well as increased sudden anger and fatigue were experienced. Important parts of life sometimes had to be put aside, such as social events and interactions, e.g., going to parties, movies, or the theater, visiting swimming pools, or going skiing, and some participants expressed the need for ideal conditions to be able to meet friends and be social. Others described loneliness due to difficulties traveling, sleeping away from home, or being a boring person who cancels social events or has a hard time finding joy due to fatigue or pain. Large social events with a lot of people were avoided by some, while others had to exclude things they wanted to do because of the pain. Further, difficulties in planning social meetings were highlighted due to uncertainty about how one would feel in the body that day.

#### 3.1.3. Illuminating the Invisible

Working hard to illuminate behaviors, symptoms, and the disease itself through extensive questioning from others such as friends, co-workers, and health professionals, increased the vulnerability and feeling of being unsuccessful in managing pain. The participants stated that since the disease is quite invisible on the outside, some people interpret their expressed inactivity, inability, or health condition as laziness or complaints. Explaining the reasons, e.g., for not participating in activities, took energy and sometimes social situations could be challenging and unsafe when met with unresponsive behaviors. The inability of others to distinguish between the participants’ normal day-to-day behavior and the painful days was perceived as a challenge to handle. Thus, some participants described a need for the disease to be highlighted for what it is to avoid misunderstandings and misinterpretations of their health condition.

### 3.2. Reaching Acceptance and Engagement

#### 3.2.1. Increasing Awareness

To be able to accept and engage in pain management, increased awareness of one’s own body limits as well as different treatments, e.g., physical activity, cold/heat, massage, medications, and altering diets such as reducing sugar, alcohol, and gluten intake, was articulated as essential, and the participants used both theoretical knowledge and lived experiences to attain this. Exploring different types of medical treatments as well as self-management strategies to decrease the pain, such as massage, heat, cold, and different oils or creams both expanded and clarified what parts of the treatment repertoire worked for each individual. For some, medical treatment was effective, either instantly, or after a longer period of testing different medications. Others had to rely on non-pharmacologic self-management strategies to handle their pain but had reached a positive acceptance of that awareness. Most participants expressed that they knew theoretically that physical activity is positive for the body and mind and used physical activity to manage the pain. Being able to move and affect different symptoms with physical activity increased their feeling of not being the disease. However, for some, moving the body and participating in different physical activities was not always associated with these positive benefits, but being aware of their own different bodily limits could motivate them to take part in suitable but not always strenuous physical activities. Thus, the importance of learning about the disease and about pain, e.g., how it is defined, whether it is dangerous, what situations or activities trigger or ease the pain, and gaining an understanding of different aspects of pain, were highlighted. Prioritizing oneself through, for example, listening to the body, practicing mindfulness, or having time alone was also expressed as useful. Altogether, the increased awareness helped the participants to feel safe in their own bodies and accept the disease.

#### 3.2.2. Finding a Permissive Environment and Social Support

Being able to land in a safe environment and using social support from family and peers were important parts of attaining acceptance by others and oneself as well as handling the pain. Many participants described their home as their castle, where there was room for just being, for making their own choices, and having the possibility of making physical adjustments, e.g., furniture. Further, the feeling of freedom and acceptance of being truly oneself was, for most participants, present at home, and the importance of close relationships, such as a partner or family, was described as vital for managing the pain. Gaining a warm and deep understanding of the disease as well as respect from others was perceived as something that could make all the difference in pain management. Being backed up by close relationships and social support was also connected to the ability to be honest, relaxed, and open about the difficulties the pain could create in daily life. However, the cognitive appraisal had taken time for some due to the inability to be honest with others about the disease. This made relationships sometimes problematic due to a feeling of being a grumpy person or due to the physical and psychological strains of carrying all the burden oneself. Others were honest right away, both to family and colleagues at work. Being honest and open about different aspects and problems related to pain as well as the fact that the disease will not go away, created closeness to others, where the possibility and willingness to share enhanced well-being. Furthermore, using peers, especially in groups, to vent different aspects of pain helped create a mutual understanding of the difficulties a life with pain can bring. For some, noticing that others have an equal or worse pain situation than oneself could create a positive change in the mind and thus create more acceptance of their own situation.

#### 3.2.3. Enhancing Inner Endurance

A strong sense of grinning and bearing in relation to inner endurance of pain in daily life elucidated the participants’ ways of accepting and handling the pain. Feelings of being able to mentally put the pain in another corner, and not think of or dig into the pain were present, and some participants also described a general enhanced tolerance for pain. However, for others, encountering the pain by really feeling and assessing it could create this sense of engagement. Some used their stubbornness from past life experiences and willingness not to feel sorry for themselves, and could thus continue with, e.g., family or work obligations despite the pain. Others also had to keep the pain experience for themselves.

### 3.3. Directing One’s Focus toward Change

#### 3.3.1. Reformulating Emotions and Thoughts

To be able to handle pain every day, using and developing emotions and thoughts was expressed as an essential strategy. Turning negative thoughts into positive ones with a feeling of being grateful for life and the things one can do, or looking for possibilities instead of problems was described as an important step in this change process. For some, instead of just feeling sick, triggering the brain to say, “I can”, could also be a successful strategy. Thus, the ability to find new ways of tackling pain or being proactive when pain accelerates compared to breaking down was present. Earlier negative thoughts of inabilities affected the psychological mood negatively but clearing the mind and instead focusing on ability made managing pain easier. Not being able to move as before due to pain, was also perceived as losing some vital part of one’s previous identity and was described as a process of grief. Learning this new way of looking at oneself included reconciling with the presence of pain, looking forward to the future, and acknowledging that this journey takes time. For some, this was expressed as a way of not letting the disease win even though the feeling of inequity could be present.

#### 3.3.2. Use of Distracting Activities

During periods of pain, different modes of distractions transformed the participants’ strength and ability to change and tone down the negative impact of pain. While the pain was present, connecting with nature or animals could enhance joy and calmness but at the same time provide a valuable alternative pain management focus. Both domestic animals and wild animals could be an important source of distraction, such as petting or listening to birdsong. Further, feeling the sun against the skin and breathing fresh air could temporarily break the bond to the pain. Some described the need for switching focus in the brain through engaging in drawing, breathing exercises, work, bodily movements, listening to music, playing a game, or participating in meditation. Others described engaging in these activities as a reality escape, where the mind must be misled. Shopping, drinking alcohol, or focusing on happy activities as well as keeping oneself occupied also provided some participants with a well-needed rest from the pain.

#### 3.3.3. Adjusted Activities

Choosing activities carefully together with a purposeful adjustment of challenging activities helped the participants to change daily life into a life where pain could be managed. Participants described, e.g., changes in household roles, purchases of domestic services, and activity pacing as important alterations at home. Furthermore, in work life, regular breaks, decreased workload or time, or working from home could also enhance this transformation. Some participants were dedicated to planning activities and social events ahead, prioritizing one thing at a time and making sure recovery was also part of the plan. It was highlighted that life is not all about work and time for physical activity but must also incorporate valuable time with family and oneself. Thus, the days were often full of activities, and altering them to fit this life with the disease was described as a challenge but doable. The need for flexibility and possibilities to make these changes in work, within the family, and in other contexts together with a dose of innovation was therefore expressed as a crucial ingredient for managing pain.

## 4. Discussion

The results of the present study provide a rich and fruitful description of the ways patients with PsA manage pain, which is a common and complex symptom of the disease. The pain is always present as a constant murmur and our results indicate that pain management with a focus on taking charge of life is developed in different ways and action components of what the patients engage in and how these actions are framed. These pain management strategies are in line with the results of a systematic review and synthesis of experiences of patients with psoriasis and PsA, describing their attempts to cope with the disease through, for example, gaining control [8] but also the strategy of keeping going despite the struggle with pain, as described by participants with chronic pain across conditions [24].

Similar aspects of pain management as described through our subtheme ‘Reaching acceptance and engagement’ have been highlighted in other studies of adults with chronic pain. Thus, a supportive ambiance has been found to be an enabler for incorporating self-management strategies in daily life [20], and a supportive environment, knowledge, and understanding facilitating factors for the self-management of pain [19]. On the other hand, barriers to self-management in cases of chronic pain have been found to include sustained motivation [20]. Thus, if managing pain as in the present study is perceived as a self-management goal, the motivation for reaching the goal needs to be approached from the perspective of a person’s different thoughts and behaviors and considering their regulation skills, as well as how personally valued the goal is. The participants described the overall approach, “Taking charge of the constant murmur of pain”, without explicitly stating whether it was a self-selected goal originating from their own intrinsic values, perceived as external pressure, or a mix of both. Self-determination theory (SDT), describing the processes through which a person acquires the motivation for health-related behaviors [21] such as the management of pain, posits that personal goal pursuit is facilitated through the satisfaction vs. frustration of one’s basic psychological needs (BPN). The BPNs are competence (feeling capable and learning different skills), autonomy (feeling that one has a choice and is in control of one’s own behavior and goals), and relatedness (feeling connected to, valued, understood, and cared for by others).

In SDT, motivation is presented as a continuum from the least autonomous, externally regulated, and controlled motivation to the intrinsically determined, autonomous motivation which depends on support for the three BPNs. Herein, we propose that the results of the present study can be interpreted in the light of SDT to help us gain further insight into and understanding of how to facilitate contextual support for BPNs in patients with PsA, to support the management of pain. The nine categories in our results describing important action components of pain management highlight the dualistic approach of both being pleased and content with the management of pain but also hesitant and somewhat unhappy. Thus, the behavior can be interpreted as partially internalized, sometimes fuller, and sometimes lesser, due to the needs satisfaction and/or frustration of BPNs. Because of this partiality together with the fluctuating pain itself, the behavior of managing pain will probably be maintained over time but with rather unstable regulation. The categories linked to the subtheme ‘Sorting out vulnerability’ are, in the light of SDT, interpreted as action components that to some extent involve the frustration of all BPNs. Furthermore, relatedness seemed to be the BNP with the lowest needs satisfaction among all categories (Table 4).

Competence satisfaction refers to the participants feeling capable of managing pain such as learning about the disease and pain itself and using different treatment strategies. Competence frustration, on the other hand, involves their feelings of doubt and failure concerning their efficacy in managing the pain, highlighted as anxiety and stress but also sometimes ignorance of pain. The feelings of competence might be compromised by long-standing pain. Dures et al. found that patients with newly diagnosed PsA need to make sense of their symptoms but also receive fast and easy access to expertise since their illness beliefs can have a large impact on them [25]. It is therefore important to give these patients timely and proper information based on their needs, as well as management tools and support to counteract, for example, prolonged avoidance of certain behaviors or not being able to ‘Sort out vulnerability’, which in turn might intensify the feeling of incompetence and decrease the motivation to attend activities for managing pain. When satisfied with the need for autonomy, the participants express a sense of personal choice and freedom in their actions such as being willing to switch focus, change thoughts, and be flexible in finding new ways of managing pain. Autonomy frustration, by contrast, involves their feelings of being forced to act in a certain way such as limiting vital daily activities or avoiding social events due to the pain. The development of a sense of autonomy is crucial for people living with PsA due to the many prevalent symptoms, including feelings of frustration that have been found in this population [26]. The subtheme ‘Directing focus to change’ might indicate that the participants in the present study had a rather well-developed autonomy in relation to adjusting and using their own activities. Still, not being able to use the body and mind all the time in a satisfactory way brings forth a wish to ‘Sort out vulnerability’ due to frustration with autonomy needs. The need for relatedness refers to the participants’ experience of genuine connection with others, like finding a safe environment, using social support, as well as gaining respect from others. Relatedness frustration involves their experience of relational exclusion and loneliness corresponding to their behavior being misinterpreted and decreased social functioning and activities. Being frustrated with being disabled by a condition not visible to others has also been found in a meta-synthesis of experiences of chronic pain across conditions [24] and might indicate the need for health professionals and peers as well as friends and family to really validate the experience of pain. Thus, relatedness can be filled with both satisfaction and frustration at the same time like in the subtheme ‘Reaching acceptance and engagement’. Experiences of flareups in PsA have been described as having an impact on several aspects of life, including social life, with refusing physical contact and social withdrawal as a consequence [27], and in rheumatoid arthritis, the social environment can have both a positive and a negative impact on pain management [28]. This indicates that pain relates to social interactions in many ways.

Since autonomous motivation for managing pain depends on the satisfaction of the three BPNs, finding a way to support these needs and thereby promote self-determined motivation is essential for patients with PsA. Autonomy support can be a successful way and refers to providing choices and options, minimizing pressure, and understanding the patient’s perspective [21]. Learning SDT-based communication skills has been found to positively influence physiotherapists’ needs-supportive behavior [29]. Another way to support the BPNs could be to provide opportunities for patients with PsA to create their own network in, for example, exercise or social activities. Stenberg et al. found that peer support interventions for the self-management of chronic pain facilitate multiple reinforcing strategies for BPNs. Another study synthesizing experiences of participating in peer-support interventions for adults with long-standing pain shows that a unique relationship can be formed between two peers, which also benefits both parties and supports BPNs [30]. Autonomy support from a spouse can also have a positive effect on the satisfaction of BPNs in people with long-standing pain [31,32].

The methodology used in the present study has some strengths and limitations. The trustworthiness of a study can be threatened by data that are not truthful or rich enough [33,34]. Thus, the interviews in the present study were performed by the first author, who was an experienced interviewer with many years as a physical therapist in rheumatology. Trustworthiness was also strengthened through the involvement in the data analysis of a researcher experienced in qualitative methods and rheumatic diseases, and peer review of the findings by a senior researcher in rheumatology. The data were also analyzed from the perspective of different professions (physical therapy and occupational therapy) [35]; however, member checks were not performed as they do not always improve research quality [36]. To attain a wide variation in the experiences of managing pain, the number of participants to be approached was not predefined. The power of purposeful sampling [37] lies in selecting cases that provide relevant, information-rich, and broad-ranging data, and through an estimate and flexible process throughout the present study, where all participants provided detailed descriptions of their experiences, the results reached a considerable depth in data. Data recruitment was stopped after 11 interviews due to data collection and analysis theme saturation [38], meaning that data replication was found, the data were rich, detailed, and relevant, and no additional information emerged in the last interviews. However, the themes and categories in the present study might not be the only ways of managing pain in PsA but highlight an understanding of different ways pain can be managed.

Because the findings must be recognizable in a clinical setting and understandable to others, relevant background data were collected for descriptive purposes. Assessment of disease activity was not collected since a widely accepted index for measuring disease activity in PsA is lacking [39]. Further, experiences of pain are not exclusively correlated to disease activity but are also related to factors such as fatigue [40], and activity limitations [41], data on which were collected. Our sample included only a few men, which might not mirror the population. Research suggests that gender can affect how an individual contextualizes and copes with pain [42]; however, in a study including men and women with rheumatoid arthritis, both genders described the same use of situation-specific strategies when managing the disease including pain [43]. In future studies, it would therefore be interesting to contrast men and women with PsA in the management of pain. Nevertheless, we believe that our findings can be recognized in and transferred to similar settings and thus be useful for health professionals seeing similar patients, both men and women.

## 5. Conclusions

Managing the constant murmur of pain in PsA by taking charge of life includes sorting out vulnerability, reaching acceptance and engagement, as well as directing focus toward change. Understanding the action components of pain management from a theoretical perspective highlights the importance for patients of satisfying three basic psychological needs: competence, autonomy, and relatedness. Health professionals, therefore, in addition to knowledge about pain and its complexity, need to stimulate autonomy by, for example, providing knowledge and choices, and supporting the patient’s own initiatives.

## Figures and Tables

**Table 1:** The main areas of the interview guide.

Main Areas of Inquiry
Times in life when pain has been highly present (challenging to manage, easy to manage)
Thoughts about reasons for pain
Factors affecting pain (personal, behavioral, social, environmental, and psychological factors)
How pain is managed
Expectations of the future in relation to pain

**Table 2:** Demographic and health-related data of the participants (n = 11).

Demographic and Health-Related Data	
Gender, female/male (n)	8/3
Age, years, md (range)	52 (40–67)
Disease duration, years, md (range)	4 (1–24)
Level of education, n	
High school	6
University	5
Occupational status, n	
Full-time work	8
Part-time work	3
Married/partner, n	10
Children at home, n	4
Pain, VAS ^1^ mm, md (range)	40 (20–70)
Fatigue, VAS ^2^ mm, md (range)	60 (0–90)
General health, VAS ^3^ mm, md (range)	70 (30–90)
Activity limitations,	
HAQ-DI ^4^, md (range), n = 9	0.875 (0–1.5)
BASFI ^5^, n = 1	2.7

^1^ Pain, VAS = visual analog scale (0–100, 0 = no pain). ^2^ Fatigue, VAS = self-perceived fatigue (0–100, 0 = no fatigue). ^3^ General health, VAS = self-perceived general health (0–100 mm (mm), 100 = excellent health). ^4^ HAQ-DI = Health Assessment Questionnaire Disability Index (0 = without difficulty, 3 = unable to do). ^5^ BASFI = Bath Ankylosing Spondylitis Functional Index (0 = without difficulty, 10 = unable to do).

**Table 3:** The steps of the data analysis.

Steps of Analysis	Authors Involved
To familiarize themselves with and obtain an overview of the material, all the transcribed individual interviews were first read carefully by one of the authors	E.S.
Open coding was performed with all the material through writing notes and headings describing approaches to pain in the text while reading it. Parts of the interviews connected to the headings were also identified. The material was read through several times while headings and related parts were identified. The headings were then moved/collected from the margins into a coding mind map. The first step of the open coding (writing notes and headings) was also performed with four of the interviews by a second author.	E.S., N.B.
The various codes were compared regarding differences and similarities. Codes with related meanings were grouped together in categories in the mind map. To ensure trustworthiness, two of the authors repeatedly discussed and compared the emerging categories with the content of the original interviews, until a negotiated consensus was reached.	E.S., N.B.
The last step of the analysis, identifying a meaningful underlying essence that runs through the material, resulted in an overarching theme of meaning and descriptive subthemes. Discussions with the author who acted as a peer expert, to compare the emerging categories and themes, were held during the final phase of the analytic procedure until a negotiated consensus was reached.	E.S., N.B., M.B.
Quotes are provided in the results for illustration and trustworthiness.	Supplementary File S1.

**Table 4:** Categories describing the action components of managing pain, interpreted as the satisfaction or frustration of basic psychological needs.

Management of Pain in PsA	Needs Satisfaction + or Frustration −		
Category in the Present Study’s Results (Action Component)	Competence	Autonomy	Relatedness
Face uncertainty for the future	−		
Consider restrictions	−	−	−
Illuminate the invisible		−	−
Increase awareness	+		
Find a permissive environment and social support		+	±
Enhance inner endurance	+	+	
Reformulate emotions and thoughts	+	±	
Use distracting activities	+	+	
Adjusted activities	+	+	

## Data Availability

Data are unavailable due to ethical restrictions.

## Supplementary Materials

The following supporting information can be downloaded at: https://www.mdpi.com/article/10.3390/jcm12237348/s1, File S1: Quotes to illustrate the result categories.
