# Supplementary material for: “It’s Like Listening to the Radio with a Little Interference”: A Qualitative Study Describing Pain Management among Patients with Psoriatic Arthritis
Source: J Clin Med. 2023 Nov 27;12(23):7348. doi: 10.3390/jcm12237348 (PMC10706874; doi:10.3390/jcm12237348)
Supplement: Supplementary file 1 [file jcm-12-07348-s001.zip › jcm-2680704-supplementary.pdf]

Supplementary File S1. Quotes to illustrate the result categories

Categories	Quote
Face uncertainty for the future	*"The fact that I have felt that it has happened, there is so much going on right now, and I kind of feel (sighs) a bit of a worry that, well, I mean, you want to have grandchildren and be able to play with them."*<br><br>*"Yes, in the beginning, I think you probably didn't pay much attention to it, but in retrospect you've understood that it's like a protective reflex kind of, the body is constantly tense because I wonder, well, what kind of pain is this, I'm not used to it. And when it tightens so much, it's just so tough, you almost become afraid of your own body because you don't know when it's going to strike."*<br><br>*"It's more that I'm afraid of what the pain does. So I think about that more than the fact that I'm actually in pain."*
Consider restrictions	*"I can get angry very quickly, explosively, and it's difficult to be. That's also a factor that has come out of this. It's painful, the children start fighting and then immediately, I used to have a lot of patience and I just get so tired and can't cope in the same way."*<br><br>*"Yeah, nowadays most people know, it doesn't affect me so much anymore, but in the past I know that it affected me quite a lot, I couldn't go away on some weekends when we had planned to do things but I was in pain and I didn't want spend the night away from home. It's still, it can still affect…just the bed, lying*

	down is very painful so I find it difficult to go away and sleep at friends' houses and that's sad."<br><br>*"When I have to watch a lecture or sit in a movie theater when I can't just get up and walk away. That has been so hard sometimes. Somewhere where you can't just take off your shoes. Or when you're sitting at work, you just sit. It's a real pain sometimes."*<br><br>*"What I don't do so often is meet my friends or go to the theater or cinema. I do that very rarely and when I do, all conditions should be right."*
Illuminate the invisible	*"Yes, but, for example, if you are going on a bus and there might not be a seat and you have to stand. And then you take a seat where it says ... yeah, you know a seat for older people…some seats. And maybe you sit there and then you can get…but people probably think like this... But they don't know that I may not be able to stand for a long time, so..."*<br><br>*"People see how it is, I mean if they are not blind. But I try to ... I often hear that ... And that can be a bit annoying, actually, when people hear that I ... It's so strange, a person who is as sick as you are and has a disease but all the time looks so lively and looks so fresh and ... But that's my way. They do not understand that it is my way of protecting myself. The tougher the day I have, the more beautiful I am."*

	And then I work at this company, where you have to exercise three hours a week, you have to have a good physique and there's also something like that.... I haven't told everyone why I don't keep up with this and that, and....you get a bit of this, are you lazy or are you comfortable or what's wrong with me (laughs)?"
Increase awareness	*"Yes, but for example alcohol, I don't drink now. I guess I have done once and then I felt that my pain and body were much worse. and body. So I have completely ruled that out. And then, exercise I think I have helped too. Maybe not immediately after, but in the long run."*<br><br>*I prefer to just lie still when I'm in pain, and not do much. But I've noticed that movement makes the pain much better, when I go and move around. Then it usually disappears."*<br><br>*Because then I have to start feeling and I have enough with just being in my own body, and sorting out pain, if it is dangerous because in the beginning, you think I can't move it's not good. But the more you understand that pain is not dangerous, the easier it has become to deal with the pain."*
Find permissive environment and social support	*"Yes, but for example at home I feel that ... there it feels positive. Yes, because my partner and my family know that I have what I have and that becomes a support in a way."*

	"I immediately talked to my whole team at work...like- this is how it is, I am, I have this disease and it looks like this and for me it will be like this and then it can change so I don't want to be asked questions every day on how I am feeling."<br><br>*"But being alone in pain is one of the worst things, I imagine. So to be in a group is important. If it then involves one or several people where you can get a silent understanding too, I think is important .... If you are with others, meet others or talk to others, without having to describe the pain, but you still get an understanding and an acceptance from those around you. I think that can be important."*
Enhance inner endurance	*"…maybe somewhere that my fighting spirit and maybe to pull myself together and not feel so sorry for myself, that has strengthened me. So I have to thank my mother for that. It's a legacy that has helped me."*<br><br>*"Of course, I've solved it like everything else I mean, but it's me who gives a lift and makes stuff happen even though I may be bedridden, I just have to get up and go. I have to, I can't: no sorry you'll have to manage, no instead it's a bit like (sighs) I bite the bullet and it has required some planning and so on which wasn't there from the beginning."*<br><br>*"I am a person who is on the move all the time. And I feel that I have probably learned to live with the pain. So I try to think the pain away. It is not possible in all situations when you may not even be able to get up on one foot, or it does*

	not want to cooperate. But as far as possible, I try to live as normally as possible."
Reformulate emotions and thoughts	*"I have always been an overachiever and it is important for me to do things, to accomplish things, to influence things. So... If I can't do that, then there's a big part of me that will be gone. And that was also the great grief when I couldn't move at all and I could not get to the car and I could not dress myself... There was a real grieving process, that the person I was... that I realized that I will never... I mean, I've always been very active, and I realized that it will never be me again. So then there was a time to mourn that, quite simply..."*<br><br>*"It's like the gratitude for what I can do, and trying to find those positive things, which I think also triggers the brain to say yes, but you actually can do it, instead of just going home and being sick."*
Use distracting activities	*"So it's something about, but just getting out and there's sun and air, it's wonderful, and then that…It's quality of life for a while to feel the heat and the air in your…it means that you don't, it's easy to get stuck in a focus on the pain too, it kind of demands your attention, which I don't want to give it. But it's easier to distract yourself."*<br><br>*"Or if you do something that catches your attention so much that you kind of… Yes, sometimes you can, yes. I'm thinking about the other day, we sat outside*

	and played TP in the sun, and it wasn't the best sitting position, but it was so much fun, cozy in the heat."<br><br>*"Yes, but if you have bought something new, then it doesn't really matter if you have a little more pain. Then it will pass. Then you don't feel ... Then something else has taken over."*
Adjusted activities	*"I've always been the kind of person who carries stuff, so now I've started ordering food from home. So I avoid all of this pulling around with these heavy carts and loading and unloading because it's really stressful. I think I've done all kinds of things over the years, but now getting the heaviest stuff delivered home makes a big difference."*<br><br>*"To find these breaks, to dare to say to yourself, now five minutes, break. It's been the hardest thing, being like a workaholic just working, working, working, and suddenly no, I need to sit down. It's super silly, but it's necessary."*<br><br>*"Well we have arranged it so that it is my husband who usually does the grocery shopping and cooking, we have also bought cleaning, we feel that it is, well, I have saved here because I can't vacuum. Because I feel that I can't, partly because my joints are so broken and unstable, so I couldn't trust my own balance."*
